# The positive impact of interprofessional education: a controlled trial to evaluate a programme for health professional students

**DOI:** 10.1186/s12909-015-0385-3

**Published:** 2015-06-04

**Authors:** Ben Darlow, Karen Coleman, Eileen McKinlay, Sarah Donovan, Louise Beckingsale, Ben Gray, Hazel Neser, Meredith Perry, James Stanley, Sue Pullon

**Affiliations:** 1 Department Primary Health Care and General Practice, University of Otago, Wellington, PO Box 7343, Wellington South New Zealand; 2 Department of Radiation Therapy, University of Otago, Wellington, PO Box 7343, Wellington South New Zealand; 3 Department of Human Nutrition, University of Otago, Wellington, PO Box 7343, Wellington South New Zealand; 4 School of Physiotherapy, University of Otago, Wellington, PO Box 7343, Wellington South New Zealand; 5 Biostatistical Group, University of Otago, Wellington, PO Box 7343, Wellington South New Zealand

**Keywords:** Controlled trial, Interprofessional education, Long-term conditions management, Pre-registration, Pre-licensure, Dietetics, Medicine, Physiotherapy, Radiation therapy, Primary health care

## Abstract

**Background:**

Collaborative interprofessional practice is an important means of providing effective care to people with complex health problems. Interprofessional education (IPE) is assumed to enhance interprofessional practice despite challenges to demonstrate its efficacy. This study evaluated whether an IPE programme changed students’ attitudes to interprofessional teams and interprofessional learning, students’ self-reported effectiveness as a team member, and students’ perceived ability to manage long-term conditions.

**Methods:**

A prospective controlled trial evaluated an eleven-hour IPE programme focused on long-term conditions’ management. Pre-registration students from the disciplines of dietetics (*n* = 9), medicine (*n* = 36), physiotherapy (*n* = 12), and radiation therapy (*n* = 26) were allocated to either an intervention group (*n* = 41) who received the IPE program or a control group (*n* = 42) who continued with their usual discipline specific curriculum. Outcome measures were the Attitudes Toward Health Care Teams Scale (ATHCTS), Readiness for Interprofessional Learning Scale (RIPLS), the Team Skills Scale (TSS), and the Long-Term Condition Management Scale (LTCMS). Analysis of covariance compared mean post-intervention scale scores adjusted for baseline scores.

**Results:**

Mean post-intervention attitude scores (all on a five-point scale) were significantly higher in the intervention group than the control group for all scales. The mean difference for the ATHCTS was 0.17 (95 %CI 0.05 to 0.30; *p* = 0.006), for the RIPLS was 0.30 (95 %CI 0.16 to 0.43; *p* < 0.001), for the TSS was 0.71 (95 %CI 0.49 to 0.92; *p* < 0.001), and for the LTCMS was 0.75 (95 %CI 0.56 to 0.94; *p* < 0.001). The mean effect of the intervention was similar for students from the two larger disciplinary sub-groups of medicine and radiation therapy.

**Conclusions:**

An eleven-hour IPE programme resulted in improved attitudes towards interprofessional teams and interprofessional learning, as well as self-reported ability to function within an interprofessional team, and self-reported confidence, knowledge, and ability to manage people with long-term conditions. These findings indicate that a brief intervention such as this can have immediate positive effects and contribute to the development of health professionals who are ready to collaborate with others to improve patient outcomes.

**Electronic supplementary material:**

The online version of this article (doi:10.1186/s12909-015-0385-3) contains supplementary material, which is available to authorized users.

## Background

Interprofessional practice is a collaborative model of healthcare which optimises the use of multiple professional skills sets to provide well-coordinated, high-quality, patient-centred care [1, 2]. Interprofessional practice requires effective communication, a clear understanding of roles and team dynamics, an ability to effectively resolve conflict, and shared leadership [2]. Communication errors are recognised as a frequent cause of adverse healthcare events and suboptimal patient care [3]. Consequently, this collaborative inter-disciplinary way of working is considered a crucial element of safe and effective care [4, 5].

Interprofessional practice is particularly important in the context of an aging population which has an increasing prevalence of long-term, complex, and co-morbid conditions. The coordinated and collaborative involvement of a team of health professionals is integral to meeting these multifaceted needs, as no single disciplinary skill set can deliver all of the care which is needed [6, 7]. Collaborative team-based care has been found to improve outcomes for people with depression [8], diabetes [9], heart failure [10], hypertension [11], terminal cancer [12], and following acute geriatric hospital admissions [13].

Interprofessional practice also has benefits for health professionals. These include increased collegial respect and trust, improved job satisfaction and role clarity, addressing negative stereotypes and professional silos, and enhanced well-being [6, 14].

Interprofessional education (IPE) is proposed as a way of improving collaborative practice and patient care [7]. It is one of the key health education reforms recommended to strengthen health systems [15]. IPE occurs when health professionals from more than one discipline learn about, from, and with each other [16]. The goal of IPE is to focus on patient-centred, team-based care through positive shared learning activities in a non-threatening environment of respectful communication and equal valuing of disciplines. This includes developing the collaborative competencies necessary for effective teamwork and gaining an appreciation of the complementary and synergistic ways different professionals can respond to patients’ needs [16, 17]. IPE aims to increase knowledge of the skills and the scopes of practice of other health professionals, to build trust, and to break down professional hierarchies which are barriers to interdisciplinary respect and trust [6, 18].

In the context of pre-registration (referred to as pre-licensure in some countries/disciplines) training, IPE aims to prepare and equip students for effective collaborative practice, however, it is often challenging to organise and implement [15]. Traditional unidisciplinary training has offered students few opportunities to interact and share learning experiences with students from other disciplines [19]. IPE is by definition an interactive learning modality, and is distinct from multidisciplinary learning approaches where students from different disciplines are simply taught side by side [20]. Undergraduate IPE interventions appear to be generally well received by students, and may increase knowledge and skills required for collaborative practice, improve student attitudes towards collaboration, and also improve clinical behaviour and patient care [1, 20–22]. There is, however, little evidence related to the maintenance of these skills over time [1].

IPE has been introduced into a number of existing health professional courses, however, this often occurs in an ad-hoc fashion whereby existing unidisciplinary programmes adopt interprofessional components [23]. While many Australasian universities report having integrated IPE into their programmes, the majority of these initiatives involve disciplines learning alongside each other and do not include the key IPE principles of learning from and about each other [16, 24]. Such learning is focused upon content rather than achieving interprofessional competencies.

The University of Otago, Wellington has developed an IPE programme over the past four years which focuses on the management of people who have long-term conditions. An uncontrolled pilot evaluation of the first year of this programme (in 2011) involving 21 students from dietetics, medicine, and physiotherapy, found that students were generally positive about the programme and that their attitudes significantly improved [22]. In 2014 this IPE programme was able to be provided to a larger cohort of students (also including the discipline of radiation therapy). This provided the opportunity to evaluate the programme using a prospective controlled study design which enabled more robust comparison between those who had and had not been exposed to the programme. This paper describes this evaluation study and its results.

The principal aim of this study was to evaluate if an IPE programme based around long-term conditions’ management changed dietetic, medicine, physiotherapy, and radiation therapy students’ attitudes to interprofessional teams, interprofessional learning, self-reported effectiveness as a team member, and perceived ability to manage long-term conditions. A secondary aim was to evaluate if students from the two larger disciplinary groups of medicine and radiation therapy responded differentially to the programme.

## Methods

This was a prospective controlled trial. The study was approved by the University of Otago Ethics Committee (D13/186).

### Participants and setting

Participants were students taking part in an IPE programme designed around providing care to people with long-term conditions taught at the University of Otago’s Wellington campus. Normal practice is for students from each disciplinary cohort to be allocated to separate groups by course administrators to meet teaching, clinical placement, and timetabling requirements. Two such groups from each discipline were able to take part in the 2014 IPE project. Those from the groups which were timetabled to receive IPE first were the intervention group, and those second were the control group. Eighty-three students were eligible to participate from the disciplines of dietetics (5^th^ and final year; *n* = 9), medicine (4^th^ year of a 6 years; *n* = 36); physiotherapy (4^th^ and final year; *n* = 12), and radiation therapy (3^rd^ and final year; *n* = 26). Allocation to intervention and control groups was performed by course administrators who were unaware of the IPE programme and its goals; the research team was not involved in group allocation. There were different numbers of students from each discipline due to the different class sizes and number of groups within each year. It was not possible to blind participants or researchers to group allocation. All intervention and control group students also continued with their usual discipline specific education throughout the period of the study.

### Intervention

The intervention involved student participation over a four-week period facilitated by an IPE-trained teaching team including faculty from dietetics, educational psychology, medicine, midwifery, nursing, physiotherapy, and radiation therapy. An initial four-hour interactive workshop included a shared meal, group discussions, small group work, Powerpoint presentations, and purpose-developed video presentations of interviews with a person living with multiple complex long-term conditions and the various health professionals involved in their management. These clips provided a basis for facilitated discussions related to living with long-term conditions, understanding patients and their context, and the nature, make-up, and role of health care teams. Students were enrolled onto a common e-learning platform to facilitate teacher communication and dissemination of instructions and resources. In interdisciplinary groups of three, students then visited a person living in the community with long-term conditions. Finally the interdisciplinary groups prepared and delivered a presentation to their peers and health professionals involved in this person’s care. This presentation included the person’s perspective of their conditions and how these are managed, together with insights as to how this may be improved by interprofessional collaboration. Following each presentation there was a group discussion facilitated by interprofessional educators to highlight aspects related to long-term condition management and interprofessional practice as well as real-world challenges associated with the care of complex patients. In total, the intervention included seven-hours of teaching contact time, and it was expected that preparing for and conducting the home visit, and then preparing the class presentation, would require an additional four hours.

### Control

**Fig. 1:**
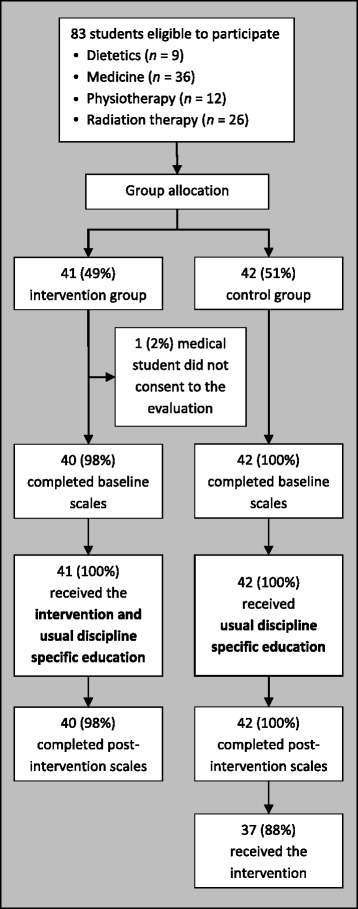
Trial flow chart

Control group students continued with their usual discipline specific education during the period of the study. These students did not receive any structured long-term condition management education or IPE during the study period, however, some students on clinical placement may have interacted with patients with long-term conditions or received clinical tutorials related to long-term conditions. The investigators were not able to control or standardise these experiences. At the completion of the study (i.e. after completing post-intervention measures), control group participants received the same IPE programme as the intervention group participants (Fig. 1).

### Measures

Student attitudes were measured pre- and post-intervention. These measures were completed during class time and at roughly equivalent time points for both intervention and control group participants (within the bounds of timetabling requirements). The time between baseline and post-intervention measures was approximately five weeks.

#### Attitudes toward interprofessional teams

These were measured with the Attitudes Toward Health Care Teams Scale (ATHCTS) [25] as modified by Curran et al. [26]. This scale has 14 items rated on a five-point Likert scale from ‘Strongly Disagree’ to ‘Strongly Agree’. Higher scores represent more positive attitudes toward teamwork [27]. The modified ATHCTS has been found to have good internal consistency when completed by health professional students [28, 29]. Three underlying constructs have been identified: ‘quality of care delivery’; ‘patient-centred care’; and ‘team efficiency’ [28].

#### Attitudes toward interprofessional learning

These were measured with the Readiness for Interprofessional Learning Scale (RIPLS) [30] as modified by Curran et al. [26]. This scale has 15 items rated on a five-point Likert scale from ‘Strongly Disagree’ to ‘Strongly Agree’. Higher scores represent more positive attitudes toward interprofessional learning. The modified RIPLS has been found to have very good internal consistency when completed by health professional students [28, 29]. Two underlying constructs have been identified: ‘expertise’; and ‘competency’ [28].

#### Perception of effectiveness as an interprofessional team member

This was measured with the Team Skills Scale (TSS) [31]. This scale has 17 items rated on a five-point Likert scale from ‘Poor’ to ‘Excellent’. Higher scores represent a higher self-reported skill level [27]. The TSS has been found to have excellent internal consistency when completed by student and graduate health professionals [32, 33].

#### Long-term condition management

The Long-term Condition Management Scale (LTCMS; see Additional file 1) was developed by the research team specifically to assess this programme because a suitable existing instrument was not identified. This research team includes experienced clinicians and researchers from a range of disciplines. The LTCMS aimed to assess whether the programme met its curricula objectives of improving students’ self-reported confidence, knowledge, and ability to manage long-term conditions. This scale has five items rated on a five-point Likert scale from ‘Completely Inadequate’ to Completely Adequate’. Higher scores represent higher self-reported confidence, knowledge, and ability. The psychometric properties of the LTCMS have not been validated to date.

### Analysis

Data were entered into a Microsoft Office Access 2010 (Microsoft Corp, Redmond, WA) database. All analyses were conducted using SPSS V20.0 for Windows software (SPSS, Inc, Chicago, Illinois, USA). Mean scores were calculated for each scale (reversed item scores were corrected before mean scale score calculation; for each scale valid responses were required to a minimum of 80 % of items to calculate a mean score). Mean attitude scale scores are reported on a 5-point scale rather than as summed scores to enable comparison between these measures which have varied numbers of items. Mean participant age and attitude scale scores (possible range 0 to 5 points) are described at baseline in intervention and control groups including 95 % confidence intervals. Other baseline characteristics (gender, ethnicity, and discipline) are summarised and compared using confidence intervals (CI) for proportions calculated using Fisher’s exact method [34]. Mean attitude scale scores post-intervention were compared between intervention and control groups using Analysis of Covariance (ANCOVA), which compared post-intervention scores adjusted for baseline scores (as a more robust analysis than comparing change scores between groups) [35]. A subgroup analysis was undertaken to assess if students from the two larger disciplinary groups of medicine and radiation therapy responded differentially to the intervention (there were insufficient dietetic and physiotherapy participants to enable meaningful comparison for these groups). Significance was judged with an alpha level of 0.05. Missing data were handled by conducting complete case analysis; only two students were missing outcomes for one or two of the scales.

## Results

Figure 1 presents the flow of participants through the study. Forty-one students were allocated to the intervention group and 42 to the control group. As all students were taking part in the IPE programme as a course requirement, irrespective of their participation in this evaluation, group allocation occurred prior to informed consent being obtained. All allocated students decided to participate in the evaluation except for one medical student, who opted out of data collection at the start of the trial (and hence no data are available for this student). Baseline and post-intervention data were collected for 40/41 intervention group participants (98 %) and 42/42 control group participants (100 %).

Baseline characteristics and attitude scale scores were comparable between intervention and control participants as can be seen from the estimates and confidence intervals (Table 1).

**Table 1:** Baseline participant characteristics and attitude scale scores

Characteristics	Intervention		Control	
	*n*	%^a^ (95%CI)	*n*	%^a^ (95%CI)
Age (years; mean)	40	21.9 (21.0,22.8)	42	22.5 (21.6,23.4)
Gender				
Female	30	75.0 (58.8,87.3)	30	71.4 (55.4,84.3)
Male	10	25.0 (12.7,41.2)	12	28.6 (15.7,44.6)
Ethnicity^b^				
NZ European	34	85.0 (70.2,94.3)	34	81.0 (65.9,91.4)
Māori	3	7.5 (1.6,20.4)	5	11.9 (4.0,25.6)
Asian	4	10.0 (2.8,23.7)	7	16.7 (7.0,31.4)
Other	0		0	
Discipline				
Dietetics	4	10.0 (2.8,23.7)	5	11.9 (4.0,25.6)
Medicine	17	42.5 (27.0,59.1)	18	42.9 (27.7,59.0)
Physiotherapy	6	15.0 (5.7,29.8)	6	14.3 (5.4,28.5)
Radiation Therapy	13	32.5 (18.6,49.1)	13	31.0 (17.6,47.1)
Scale (mean score^c^)				
ATHCTS	39^d^	3.9 (3.8,4.0)	42	3.8 (3.7,3.9)
RIPLS	40	3.9 (3.8,4.1)	42	3.9 (3.8,4.0)
TSS	40	2.9 (2.7,3.1)	42	3.0 (3.8,3.1)
LTCMS	40	3.1 (2.9,3.3)	42	2.9 (2.8,3.1)

*NZ* New Zealand, *ATHCTS* Attitudes Toward Health Care Teams Scale, *RIPLS* Readiness for Interprofessional Learning Scale, *TSS* Team Skills Scales, *LTCMS* Long-Term Condition Management Scale

^a^unless otherwise specified

^b^total equals more than 100 % as respondents were able to select more than one category

^c^scored on 5-point Likert scale, higher scores represent more positive attitudes

^d^one student provided insufficient valid responses to allow a mean to be calculated for the ATHCTS

**Fig. 2:**
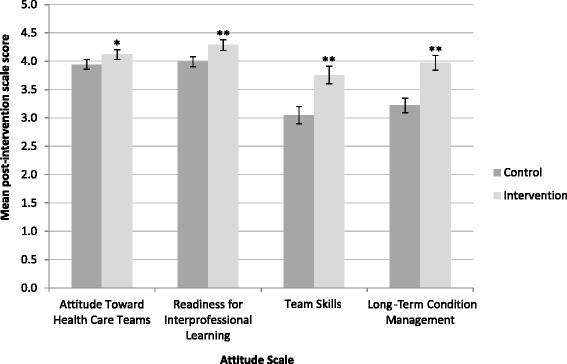
Mean post-intervention scale scores in control and intervention group participants adjusted for baseline scores. Error bars represent 95 % confidence intervals. * *p* < 0.05; ** *p* < 0.001. Analysis for the ATHCTS was missing data for one student and analysis for the TSS was missing data for two students due to insufficient valid responses being provided

Mean post-intervention attitude scores (on a five-point scale) adjusted for baseline scores were significantly higher in the intervention group than the control group for all scales (Fig. 2). The mean difference for the ATHCTS was 0.17 (95 %CI 0.05 to 0.30; *p* = 0.006), for the RIPLS was 0.30 (95 %CI 0.16 to 0.43; *p* < 0.001), for the TSS was 0.71 (95 %CI 0.49 to 0.92; *p* < 0.001), and the LTCMS 0.75 (95 %CI 0.56 to 0.94; *p* < 0.001). Analysis for the ATHCTS was missing data for one student and analysis for the TSS was missing data for two students due to insufficient valid responses being provided.

The mean effect of the intervention was similar for students from the two larger disciplinary sub-groups of medicine and radiation therapy (Table 2).

**Table 2:** Mean post-intervention scale scores adjusted for baseline scores in medicine and radiation therapy students

Scale	Discipline	Intervention		Control	
		*n*	Mean score^a^ (95%CI)	*n*	Mean score (95%CI)
ATHCTS	Medicine	16^b^	4.1 (4.0,4.3)	18	4.0 (3.8,4.1)
	Radiation therapy	13	4.1 (3.9,4.3)	13	3.8 (3.7,4.0)
RIPLS	Medicine	17	4.3 (4.1,4.4)	18	3.9 (3.8,4.1)
	Radiation therapy	13	4.2 (4.0,4.3)	13	3.9 (3.7,4.0)
TSS	Medicine	15^c^	3.8 (3.5,4.0)	18	3.2 (3.0,3.4)
	Radiation therapy	13	3.8 (3.5,4.1)	13	2.9 (2.6,3.1)
LTCMS	Medicine	17	4.0 (3.8,4.2)	18	3.0 (2.8,3.2)
	Radiation therapy	13	4.0 (3.7,4.2)	13	3.3 (3.1,3.5)

*ATHCTS* Attitudes Toward Health Care Teams Scale, *RIPLS* Readiness for Interprofessional Learning Scale, *TSS* Team Skills Scales, *LTCMS* Long-Term Condition Management Scale

^a^Mean post-intervention score adjusted for baseline score. Scored on 5-point Likert scale, higher scores represent more positive attitudes

^b^one student provided insufficient valid responses to allow a baseline mean to be calculated for the ATHCTS

^c^two students provided insufficient valid responses to allow a post-intervention mean to be calculated for the TSS

## Discussion

### Summary of findings

This prospective controlled trial found significant improvement in students’ attitudes towards both interprofessional teams and interprofessional learning as a result of receiving an eleven-hour IPE intervention. It also found significant improvements in intervention group students’ self-reported effectiveness as a team member and self-perceived confidence, knowledge, and ability to manage long-term conditions.

### Strengths and limitations

The experimental design enabled robust analysis of changes in attitudes of those exposed and not exposed to the intervention. No participants were lost to follow-up and complete data were available for almost all participants. Although group allocation was not random, the process of allocating students to groups was completely independent of the study hypothesis or outcomes. This is similar to the approach taken in an IPE study by Street et al. [36]. No baseline differences were identified between intervention and control groups. The participants in this study were predominantly young, female, and of New Zealand European ethnicity. This is reflective of those participating in undergraduate health professional education in New Zealand, but may limit the ability to generalise these findings to other populations. The sample size for this study was determined based upon resource and student availability rather than a formal power calculation. Confidence intervals for estimates (differences in outcome scale means between groups) provide a metric for considering the impact of sample size on the statistical precision of the results, and these confidence intervals were generally narrow.

The control group participants did not receive the same long-term conditions’ management content as the intervention group participants. Consequently, it is not possible to say if the changes seen in the intervention group are solely due to the interprofessional nature of the intervention, or if they are also related to its curricula content. In addition, the educational experiences received as part of students’ usual discipline specific education outside of the intervention were not uniform for all participants; it is possible that these may have also influenced their attitudes. Randomising students in each of the sequential teaching groups to receive long-term conditions’ management training in either interprofessional or unidisciplinary environments may have addressed these issues, but it was not logistically feasible to provide different teaching to half of each group, nor was it ethically acceptable to deny half the group an opportunity to participate in order to improve the rigour of evaluation.

The three scales used to measure attitudes toward interprofessional teams and learning, and self-reported effectiveness as a team member, have previously been validated. The use of these commonly employed instruments improves the ability to compare these results with other studies and synthesise IPE evidence [21, 37]. Despite this, the correlation between these scales and clinical behaviour or treatment outcome is unknown. Although the magnitude of these changes appears to be large, particularly with respect to effectiveness as a team member, it is not known if these changes are clinically meaningful. The fact that the IPE programme was provided to the control group participants at the completion of the study also means that the long-term effects of the intervention are not able to be studied.

The LTCMS was developed for this study to enable assessment of the intervention’s impact on the curricula objective of improving students’ knowledge, confidence, and ability to manage long-term conditions. This is the first reported use of this instrument and its psychometric properties are yet to be established.

### Comparison with previous research

The baseline ATHCTS and RIPLS scores for students in this study were almost identical to those found in a large Canadian sample of medical students [29], and an Australian sample including dietetic, medical, physiotherapy, and radiation therapy students [38].

The authors are only aware of three previous randomised controlled trials which have evaluated pre-registration IPE interventions [36, 39, 40]. Similar to the current study, two of these studies based their interprofessional teaching around content involving long-term conditions’ management [36, 40], with one of these also involving a student visit to a person’s home and subsequent peer presentation [36]. Although each of these three studies found some small benefits related to IPE for at least one of the outcome measures used, none have used standardised outcome measures, nor have they evaluated the effect of IPE on more than two disciplines. Consequently, results are difficult to compare with those of the current study.

An uncontrolled evaluation of a pre-registration IPE programme which also involved six-hours of teacher contact time by Wellmon et al. [41] found a similar magnitude of change on the ATHCTS (0.19 versus 0.17; note these and subsequent scores from other papers have been rescaled to a 5-point scale to enable comparison with the current study’s results), but a smaller change on the RIPLS than the current study (0.15 versus 0.30). Similarly, Wakely et al. [38] found a small change in RIPLS scores (0.21) following a four and a half hour IPE intervention. In comparison, a nine-hour IPE programme for *graduate* primary care professionals from seven disciplines found that ATHCTS scores did not change, and TSS scores only increased by a small amount (0.14 versus 0.71 for the current study) [27]. This different finding may indicate that the design of the intervention in the current study improved team skills more effectively, or that pre-registration students’ attitudes are less well-formed and therefore more amenable to change. Interestingly, qualitative comments from participants in the study by Robben et al. [27] indicated that despite the limited change in attitude scale scores, participants valued the programme’s interprofessional nature and felt it had increased their collaboration with other professionals. Participants also felt that their knowledge of other professions and understanding of their viewpoints had increased [27]. It could be hypothesised therefore that the degree of attitude change found in the current study could correlate with marked changes in collaborative behaviour.

The intervention in the current study contained many different elements. These include the facilitation of learning by an experienced team of interprofessional educators who have a range of disciplinary backgrounds, the use of purpose-designed media to aid learning, a requirement for interdisciplinary groups of students to independently arrange and conduct an interview with someone who has one or more long-term conditions in this person’s home, unstructured interaction between students, and peer presentations with facilitated discussion. The current study is not able to quantify the relative importance or effect of each of these elements. Previous qualitative analysis of a pilot intervention indicated that students perceived social elements of the programme and interactions with each other as being of particular importance [22].

### Implications for health professional education and future research

The findings of this study indicate that the IPE programme met both its interprofessional objectives and its subject-based learning objectives. This suggests that even short IPE interventions using purpose-developed resources can be effective in fostering positive attitudes towards working interprofessionally. Furthermore, it is possible that embedding IPE learning within the context of a relevant topic like long-term conditions’ management, and using patient-centred approaches such as a home visit, may enhance students’ learning and their ability to anticipate plausible clinical scenarios, thereby translating IPE principles into ‘real world’ thinking. These potential explanations require further exploration using qualitative methodologies and prospective, long-term studies.

This IPE programme has now been established at the University of Otago, Wellington for a number of years [22]. This has enabled the development of an established teaching team who have gained skills in the delivery of IPE and this may have enhanced the effect of the intervention. Both the programme and the teaching team were adapted to facilitate the integration of students and faculty from a new discipline. The similar degree of attitude change across the disciplinary groups supports the inclusion of students from a range of disciplines and suggests that additional disciplines can be added to existing IPE programmes.

Process evaluations of future IPE programmes may help to clarify which elements have the most influence on learning outcomes so that these may be optimised. These elements may include the learning approaches, disciplinary mix and experience of teachers and students, the curricula content around which learning is based, and the balance between structured and unstructured interaction. Future studies are also required to establish if the effect of such short-term interventions is maintained over time, and importantly if these effects influence future clinical behaviour and patient outcomes. Very few data are available regarding the translation of collaborative attributes and competencies acquired during pre-registration IPE courses into clinical practice [37]. One of the few longitudinal studies of the impact of pre-registration IPE on health professionals after graduation found sustained increased confidence relating to participants’ communication skills and increased positive attitudes toward interprofessional relationships [42]. This study did not evaluate clinical behaviour or patient outcomes. It is generally accepted that trying to evaluate these long-term outcomes following short-term pre-registration IPE programmes is not feasible due to the wide range of potential confounding factors [37]. Rather, these types of programmes are considered to be an investment in the future [37]. Regular exposure to IPE throughout pre-registration training may help to embed collaborative practice as an integral part of health professional practice [15].

## Conclusions

An eleven-hour interprofessional education programme resulted in improved attitudes toward interprofessional teams and interprofessional learning for dietetic, medicine, physiotherapy, and radiation therapy students. It also resulted in improved self-reported effectiveness as an interprofessional team member, and self-perceived confidence, knowledge, and ability to manage people with long-term conditions. These findings indicate that a brief intervention about an appropriate subject can have positive effects and contribute to the development of health professionals who are ready to collaborate with others in order to improve patient outcomes.

## Additional file

Additional file 1: **The Long Term Condition Management Scale.** Scale developed by the research team to measure self-reported confidence, knowledge, and ability to manage long-term conditions.

## Abbreviations

IPE: Interprofessional education
ATHCTS: Attitudes Toward Health Care Teams Scale
RIPLS: Readiness for Interprofessional Learning Scale
TSS: Team Skills Scale
LTCMS: Long-Term Condition Management Scale
95%CI: 95 percent confidence interval

## References

[1] Lapkin S, Levett-Jones T, Gilligan C (2013). A systematic review of the effectiveness of interprofessional education in health professional programs. Nurse Educ Today.

[2] Canadian Interprofessional Health Collaborative (2010). A national interprofessional competency framework.

[3] Leonard M, Graham S, Bonacum D (2004). The human factor: the critical importance of effective teamwork and communication in providing safe care. Qual Saf Health Care.

[4] Oandasan I, Barker K, Barker C, Bosco D, D’Amour L, Jones S, Kimpton L, Lemieux-Charles L, Nasmith IL, San Martin Rodriguez J (2006). Teamwork in healthcare: promoting effective teamwork in health care in Canada.

[5] Timmel J, Kent PS, Holzmueller CG, Paine L, Schulick RD, Pronovost PJ (2010). Impact of the Comprehensive Unit-based Safety Program (CUSP) on safety culture in a surgical inpatient unit. Jt Comm J Qual Patient Saf.

[6] Hall P (2005). Interprofessional teamwork: professional cultures as barriers. J Interprof Care.

[7] Thistlethwaite J (2012). Interprofessional education: a review of context, learning and the research agenda. Med Educ.

[8] Unützer J, Katon W, Callahan CM, Williams JW, Hunkeler E, Harpole L, Hoffing M, Della Penna RD, Noël PH, Lin EH (2002). Collaborative care management of late-life depression in the primary care setting: a randomized controlled trial. JAMA.

[9] Aubert RE, Herman WH, Waters J, Moore W, Sutton D, Peterson BL, Bailey CM, Koplan JP (1998). Nurse case management to improve glycemic control in diabetic patients in a health maintenance organizationA randomized, controlled trial. Ann Intern Med.

[10] McAlister FA, Stewart S, Ferrua S, McMurray JJ (2004). Multidisciplinary strategies for the management of heart failure patients at high risk for admission: a systematic review of randomized trials. J Am Coll Cardiol.

[11] Carter BL, Rogers M, Daly J, Zheng S, James PA (2009). The potency of team-based care interventions for hypertension: a meta-analysis. Arch Intern Med.

[12] Higginson IJ, Evans CJ (2010). What is the evidence that palliative care teams improve outcomes for cancer patients and their families?. Cancer J.

[13] Deschodt M, Flamaing J, Haentjens P, Boonen S, Milisen K (2013). Impact of geriatric consultation teams on clinical outcome in acute hospitals: a systematic review and meta-analysis. BMC Med.

[14] Mickan SM (2005). Evaluating the effectiveness of health care teams. Aust Health Rev.

[15] Frenk J, Chen L, Bhutta ZA, Cohen J, Crisp N, Evans T, Fineberg H, Garcia P, Ke Y, Kelley P (2010). Health professionals for a new century: transforming education to strengthen health systems in an interdependent world. Lancet.

[16] World Health Organisation (2010). Framework for action on interprofessional education and collaborative practice.

[17] Interprofessional Education Collaborative Expert Panel (2011). Core competencies for interprofessional collaborative practice: Report of an expert panel.

[18] Oandasan I, Reeves S (2005). Key elements of interprofessional education. Part 2: factors, processes and outcomes. J Interprof Care.

[19] Barr H, Koppel I, Reeves S, Hammick M, Freeth D (2005). Effective interprofessional education: argument, assumption and evidence.

[20] Hammick M, Freeth D, Koppel I, Reeves S, Barr H (2007). A best evidence systematic review of interprofessional education: BEME Guide no. 9. Med Teach.

[21] Reeves S, Goldman J, Burton A, Sawatzky-Girling B (2010). Synthesis of systematic review evidence of interprofessional education. J Allied Health.

[22] Pullon S, McKinlay E, Beckingsale L, Perry M, Darlow B, Gray B, Gallagher P, Hoare K, Morgan S (2013). Interprofessional education for physiotherapy, medical and dietetics students: a pilot programme. J Prim Health Care.

[23] Payler J, Meyer E, Humphris D (2008). Pedagogy for interprofessional education–what do we know and how can we evaluate it?. Learn Health Soc Care.

[24] Gilligan C, Outram S, Levett-Jones T (2014). Recommendations from recent graduates in medicine, nursing and pharmacy on improving interprofessional education in university programs: a qualitative study. BMC Med Educ.

[25] Heinemann GD, Schmitt MH, Farrell MP, Brallier SA (1999). Development of an attitudes toward health care teams scale. Eval Health Prof.

[26] Curran VR, Sharpe D, Forristall J (2007). Attitudes of health sciences faculty members towards interprofessional teamwork and education. Med Educ.

[27] Robben S, Perry M, van Nieuwenhuijzen L, van Achterberg T, Rikkert MO, Schers H, Heinen M, Melis R (2012). Impact of interprofessional education on collaboration attitudes, skills, and behavior among primary care professionals. J Contin Educ Health.

[28] Hayashi T, Shinozaki H, Makino T, Ogawara H, Asakawa Y, Iwasaki K, Matsuda T, Abe Y, Tozato F, Koizumi M (2012). Changes in attitudes toward interprofessional health care teams and education in the first- and third-year undergraduate students. J Interprof Care.

[29] Curran VR, Sharpe D, Forristall J, Flynn K (2008). Attitudes of health sciences students towards interprofessional teamwork and education. Learn Health Soc Care.

[30] Parsell G, Bligh J (1999). The development of a questionnaire to assess the readiness of health care students for interprofessional learning (RIPLS). Med Educ.

[31] Hepburn K, Tsukuda RA, Fasser C, Heinemann GD, Zeiss AM (2002). Team skills scale. Team performance in health care: assessment and development.

[32] Curran V, Heath O, Adey T, Callahan T, Craig D, Hearn T, White H, Hollett A (2012). An approach to integrating interprofessional education in collaborative mental health care. Acad Psychiatry.

[33] Fulmer T, Hyer K, Flaherty E, Mezey M, Whitelaw N, Jacobs MO, Luchi R, Hansen JC, Evans DA, Cassel C (2005). Geriatric interdisciplinary team training program evaluation results. J Aging Health.

[34] Armitage P, Berry G, Matthews J (2002). Statistical methods in medical research.

[35] Frison L, Pocock SJ (1992). Repeated measures in clinical trials: analysis using mean summary statistics and its implications for design. Stat Med.

[36] Street KN, Eaton N, Clarke B, Ellis M, Young PM, Hunt L, Emond A (2007). Child disability case studies: an interprofessional learning opportunity for medical students and paediatric nursing students. Med Educ.

[37] Reeves S, Perrier L, Goldman J, Freeth D, Zwarenstein M (2013). Interprofessional education: effects on professional practice and healthcare outcomes (update). Cochrane Database Syst Rev.

[38] Wakely L, Brown L, Burrows J (2013). Evaluating interprofessional learning modules: health students’ attitudes to interprofessional practice. J Interprof Care.

[39] Just JM, Schnell MW, Bongartz M, Schulz C (2010). Exploring effects of interprofessional education on undergraduate Students’ behaviour: a randomized controlled trial. J Res Interprof Pract Educ.

[40] Nango E, Tanaka Y (2010). Problem-based learning in a multidisciplinary group enhances clinical decision making by medical students: a randomized controlled trial. J Med Dent Sci.

[41] Wellmon R, Gilin B, Knauss L, Inman Linn M (2012). Changes in student attitudes toward interprofessional learning and collaboration arising from a case-based educational experience. J Allied Health.

[42] Pollard KC, Miers ME (2008). From students to professionals: results of a longitudinal study of attitudes to pre-qualifying collaborative learning and working in health and social care in the United Kingdom. J Interprof Care.

