# Supplementary material for: The positive impact of interprofessional education: a controlled trial to evaluate a programme for health professional students
Source: BMC Med Educ. 2015 Jun 4;15:98. doi: 10.1186/s12909-015-0385-3 (PMC4462076; doi:10.1186/s12909-015-0385-3)
Supplement: Additional file 1: — The Long Term Condition Management Scale. Scale developed by the research team to measure self-reported confidence, knowledge, and ability to manage long-term conditions. [file 12909_2015_385_MOESM1_ESM.docx]

Please answer all questions **#** Mark your answers like this If you make a mistake, do this

then tick the correct response

**THE LONG TERM CONDITION MANAGEMENT SCALE**

Completely inadequate

Completely adequate

1. **I think my confidence to talk with people with long-term conditions about how their care is organised is:**
2. **I think my confidence to talk with people with long-term conditions about their ability to self-manage is:**
3. **I think the knowledge I have about organising care for people with long-term conditions is:**
4. **I think my knowledge of what other disciplines can contribute to long-term conditions care is:**
5. **I think the ability I have to work collaboratively with other disciplines to organise care for people with long-term conditions is:**
